# A Suggested New Departure in Hospital Administration

**Published:** 1904-12-10

**Authors:** 


### HOSPITAL ADMINISTRATION.

### CONSTRUCTION AND ECONOMICS.

### A SUGGESTED NEW DEPARTURE IN HOSPITAL ADMINISTRATION.

### III. THE MEDICAL SUPERINTENDENT—AN INDISPENSABLE OFFICIAL.

Having dealt with the advantages and disadvantages attaching to the proposal to place a large general hospital with hundreds of beds under the control of a chairman-superintendent, we will now explain why we consider a medical superintendent in charge of every department of a great hospital to be an indispensable official.

It must be understood that the medical superintendent of a great hospital has no responsibility for the treatment of the patients after they are once admitted into the wards. The work of the medical superintendent is mainly administrative. Every well-regulated hospital endeavours to control, with infinite care, the admission of in-patients. To effect this it is desirable to fix an hour of admission each day when all the new cases must apply, so that the medical superintendent may be in attendance during the whole of the admission hours, may supervise the work of the juniors entrusted with the duty of examining the cases, and decide in all cases of doubt whether a particular patient is to be admitted or not. There remain, of course, the urgent cases which are sent to the hospital throughout the day and evening and sometimes during the night. The casualty department should be so organised and placed that the patients on their arrival can be taken to it that they may be examined, and where the decision as to admission or refusal should be determined, after examination by a resident when necessary, by reference to the medical superintendent or his assistant. In this way, providing there are a few emergency beds for "drunk or dying" and noisy cases in contiguity to the casualty department, it should be impossible for any hospital to suffer discredit or criticism in regard to the admission arrangements for patients. Wherever there is no medical superintendent scandals must arise, but where the system is properly organised under the medical superintendent, providing this officer knows his duty and does it, the reputation of the hospital must be safeguarded. As soon as a patient is admitted to a ward he is placed in charge of one of the honorary medical staff, who thenceforward will be responsible for his retention in or discharge from the hospital.

Taking next the enforcement of economy and the prevention of waste, the superintendent's duties are not difficult to define. He will make it his business to frequently visit the wards, and every department of the institution, for in no other way can he exercise a general superintendence and control over the whole establishment. He will provide that no orders are issued except in writing, and that no goods are admitted to the hospital without his written order. The whole of the orders issued being directly under the control of the medical superintendent, he will arrange to have all the various stores of every kind reduced to their cash values and a monthly account kept of the actual issues of every article and its value, so that it may be possible for him to tell at once when the expenditure upon any item shows an abnormal increase, to promptly investigate the causes which have led to this, and to decide whether such increase is justifiable or preventible. For his own purposes, if he be the right man in the right place, he will probably keep a daily return of the issues of every article used in the hospital, for in this way it is possible for an efficient officer to keep the expenditure practically uniform, or nearly so, from year to year. The great evil of modern hospitals is, in practice, the continuous tendency to increase the cost of all departments.

We have been at pains to test, for the purposes of these articles, the efficiency, in practice, of the system here advocated. We find that in a great hospital having upwards of 6,000 in-patients a year it has been possible during the years 1903 and 1904 to so control the whole establishment and the expenditure on every item, that in the matter of provisions, although the in-patients have been slightly greater in number in 1904 than in 1903, the total outlay in 1904 on provisions has decreased by upwards of £50. When we examine the items in detail, we find that there has been a saving of nearly £100 on butchers' meat, whereas there has been an increase of nearly £50 upon bread, owing to the rise in price. The consumption of alcohol has been reduced by upwards of £20, whereas there has been an increase in sugar, owing to the rise in prices, of about the same amount. It is interesting to note in connection with the decrease in butchers' meat that the number of eggs consumed has increased in 1904 to the value of £35 for the twelvemonths. Dealing with all the other items of expenditure upon supplies of various kinds, including uniforms and the wages paid to artisans on the permanent staff, a net saving in expenditure of £587 has been effected during 1904 compared with 1903. The largest item is the saving on coals owing to a fall in prices, which represents about two-fifths of the whole saving. On 36 items a saving is shown upon two out of every three. One other advantage of this control is, that by supplying each member of the honorary medical staff with a return showing the expenditure upon the medical supplies used for his patients each month, it has been possible to secure the co-operation of the surgeons, especially, with the result, that the average expenditure per annum upon the beds of each of five surgeons approximates very closely to the average of the whole.

The tables are very interesting, and we hope on another occasion to publish the figures in detail. They prove to demonstration that the majority of hospitals in London do not at present possess a system which secures effective control over every item of expenditure in a form which effectually checks every upward tendency, and stops it promptly, unless it can be proved to be due to causes which are special or justifiable. The committee of every London hospitals should make it their business to devote a few meetings to the discussion of this question, and the establishment of this system of effective control. One of the best administered hospitals outside London with a large medical school attached, can provide everything for the in-patients, including clothing and personal linen during their residence in the hospital, for about £70 per occupied bed, whereas several of the principal London hospitals, which do not provide anything like the same articles, expend £120 and upwards per occupied bed year by year. We hope that the King's Fund and the Hospital Sunday Fund will exercise their influence to bring about reforms which must tend to improve the efficiency of the working of the majority of metropolitan hospitals by keeping their expenditure within reasonable limits.

Then, too, the control exercised by the medical superintendent promotes the comfort of everybody in the house. Discipline is maintained, justice is enforced, comfort is promoted, and friction is avoided. It is not fair, for instance, to blame a junior medical officer without experience for mistakes occurring in regard to his handling of patients applying for admission to a hospital if he has not behind him the support and counsel of an experienced medical superintendent. Indeed, we are inclined to feel that where a hospital is attached to a medical school the absence of a medical superintendent is a great injustice to the junior medical officers, for it deprives them of the counsel and experience to which they are entitled at the hands of the hospital authorities, and these can alone be supplied by an efficient medical superintendent. The constant presence of a medical superintendent within call secures uniformity in the practice of each department, a fact which makes for economy and efficiency. It is a great matter that every member of the staff should feel that the medical superintendent understands his duties, that he is constantly moving about, that he observes every omission or departure from the rules, and that carelessness or inefficiency will be promptly noted. The wonder is that anybody who thinks about the matter at all can suppose, that a great institution can work smoothly, or that the responsibilities it owes to the public can be duly and continuously fulfilled, in the absence of a general superintendence and control of the whole establishment by a medical superintendent, possessed of an intimate personal knowledge of every detail connected with the work of each department. There is a marked absence of any such control at most of the London hospitals, and the time is not far distant, if it has not already arrived, when the present system must be changed and economy everywhere enforced.

---

The foundation-stone has been laid of an accident hospital at Fullerton. This hospital will be close to Denaby Main, where thousands of miners work in the Denaby and Cadeby Pits. The first portion to be completed will contain from six to eight beds. The workmen have already contributed £933 to the scheme, and their representative has been elected to act as a trustee.

